# Stand spatial structure promotes tree growth and sapling diversity in northern tropical karst seasonal rainforest

**DOI:** 10.3389/fpls.2025.1649999

**Published:** 2025-12-03

**Authors:** Lingyan Li, Ruixia Ma, Bin Wang, Fuzhao Huang, Jianxing Li, Fang Lu, Wusheng Xiang, Dongxing Li, Xiankun Li, Yili Guo

**Affiliations:** 1 Guangxi Key Laboratory of Environmental Processes and Remediation in Ecologically Fragile Regions, Guangxi Normal University, Guilin, Guangxi, China; 2 Guangxi Key Laboratory of Plant Conservation and Restoration Ecology in Karst Terrain, Guangxi Institute of Botany, Guangxi Zhuang Autonomous Region and Chinese Academy of Sciences, Guilin, Guangxi, China; 3 Nonggang Karst Ecosystem Observation and Research Station of Guangxi, Chongzuo, Guangxi, China

**Keywords:** stand spatial structure, sapling diversity, nearest-neighbor trees, dynamic changes, topography, forest dynamic plot, karst rainforest

## Abstract

Stand spatial structure and sapling diversity are essential for maintaining ecosystem stability. However, the dynamic characteristics of stand spatial structure, its driving factors, and its influence on sapling diversity remain unclear. This study was conducted in a 15-ha forest dynamics plot located in the seasonal rainforest of the northern tropical karst region. We analyzed stand dynamics by characterizing multivariate distributions of stand spatial structure within random structural units, together with stand growth, mortality, and recruitment processes. To evaluate spatial autocorrelation and its drivers, we constructed spatial lag models and spatial error models. Generalized additive models were further applied to assess the effects of topography on stand spatial structure, as well as the influence of stand spatial structure on sapling diversity. The overall stand exhibited a uniform angle index of 0.5, a mingling index of 0.75, and a dominance index of 0.49, indicating random species distribution with moderate to high mingling. Null, univariate, bivariate, and trivariate distributions of stand spatial structure exhibited no significant change over the past decade. At the individual-tree level, however, survival, mortality, and recruitment processes induced notable shifts in spatial structure, which were more pronounced than the overall stand-level dynamics. Topography strongly influenced spatial structural metrics: elevation explained 31.35% and 64.99% of the variance in mingling and dominance, respectively, making it the most important factor for these indices, while slope accounted for 22.53% of the variance in the uniform angle index, serving as its primary driver. Among structural attributes, mingling had overwhelming explanatory power for sapling diversity, accounting for 99.68% of the variance in the Shannon–Wiener index, 99.70% in the Simpson index, 54.88% in Pielou’s evenness index, and 99.69% in Margalef’s diversity index, identifying it as the dominant factor regulating sapling diversity. These findings demonstrate that considering the dynamic changes of stand spatial structure at the individual-tree level, together with its effects on sapling diversity, is essential for understanding the structural and functional properties of tropical karst forests.

## 1 Introduction

Stand spatial structure refers to the distribution patterns of individual trees and the arrangement of their attributes (Chai et al., 2017). It reflects both facilitative and competitive interactions among trees (Nguyen et al., 2022) and strongly influences forest ecological processes and overall ecosystem health (Zhang et al., 2024). Moreover, stand spatial structure and tree growth are reciprocally linked (Pommerening, 2006). With the full implementation of the Natural Forest Protection Program, research on planted forest management and forestry industry has expanded considerably. However, natural forests continue to provide greater ecological benefits than planted forests, making their protection and development a long-standing research priority (Farooq et al., 2021, 2020, 2019). Therefore, comprehensive investigations of stand spatial structure are essential for optimizing silvicultural practices and advancing sustainable forest management.

Over the past several decades, numerous methods have been developed to quantify the stand spatial structure attributes. Increasingly, however, finer-scale analyses have become essential. The relationships between individual tree and their nearest neighbors are particularly crucial for understanding competition over limited resources, mutual dependence, and species coexistence (Gadow, 1998). Among the most widely used parameters for describing stand spatial structure are the uniform angle index (W) (Hui and Gadow, 2003), mingling index (M) (Gadow and Füldner, 1993), and dominance index (U) (Gadow, 1998), all of which are derived from the characteristics of adjacent trees. These indices are frequently applied to analyze community structure attributes (Zhang et al., 2018). The uniform angle index evaluates the spatial distribution uniformity between a reference tree and its four nearest neighbors by comparing the observed angles to the theoretical standard of 72°. The dominance index quantifies species dominance within a stand by measuring the proportion of neighboring trees that are larger or smaller than the reference tree. The mingling index measures species segregation by calculating the proportion of neighboring trees belonging to species different from the reference tree, thereby reflecting the degree of spatial mixing among tree species. Compared with traditional stand-level methods, analyses based on structural units of adjacent trees provide a more detailed description of local configuration and microenvironment (Aguirre et al., 2003). The approach is strongly linked to spatial ecological niches, the growth potential of surrounding trees, and the overall stability of forest ecosystems (Dong et al., 2022; Yang et al., 2019).

To date, extensive analyses of stand spatial structure have been carried out by numerous scholars. These studies have examined univariate, bivariate and trivariate distributions, thereby deepening the understanding of stand structure attributes (Wang et al., 2023; Yang et al., 2019). However, stand spatial structure is a dynamically evolving process (Lian et al., 2024; Zhang et al., 2022), primarily shaped by tree growth, mortality, and recruitment. Regeneration and mortality strongly affect the distribution and competitive interactions of surrounding trees, thereby influencing the spatial structure of stands. Many previous studies have emphasized tree growth, while often neglecting the critical roles of mortality and regeneration. As a result, the collective influence of mortality and regeneration on stand spatial structure remains poorly understood. Stand spatial structure represents an outcome of long-term ecological succession, reflecting adaptation to the surrounding environment. At the same time, microenvironmental conditions within stands, including light intensity, temperature distribution, and soil moisture, also regulate stand spatial structure. These interaction form complementary and dynamically balanced ecological processes. To clarify the dynamic changes in stand spatial structure and their underlying causes, detailed analyses of the driving factors are essential. Recent studies have identified stand conditions, soil properties, topographic features, and thinning intensity as key influences on spatial structure (Wang et al., 2024; Zhang et al., 2020). Nevertheless, investigations of karst natural forests that simultaneously integrate both biotic and topographic factors remain scarce. In northern tropical karst seasonal rainforests, topographic factors, especially elevation, have been shown to strongly influence tree habitat partitioning, species diversity, and community composition (Guo et al., 2019, 2017a). Given the reciprocal effects of species composition, diversity, and habitat conditions on stand spatial structure, we hypothesize that variation in elevation in these forests creates distinct topographic habitats, which in turn shape species composition and distribution patterns, thereby altering stand spatial structure.

Stand spatial structure is also shaped by biotic factors. The mingling-size hypothesis suggests that large trees are typically surrounded by smaller, heterospecific neighbors, resulting in a positive correlation between local species mingling and size inequality (Wang et al., 2021). Tree size is therefore considered a primary factor influencing spatial interactions. However, whether mingling and dominance are consistently size dependent remains unclear. Larger trees often homogenize their local spatial distribution, whereas the clustered growth of smaller individuals may reflect resource competition or regeneration strategies. Dominance is generally negatively correlated with tree size, yet in karst regions, the relationship between tree size and stand spatial structure indices remains poorly understood. Furthermore, the interactions between additional biotic factors, such as individual plant density and total basal area at breast height, and stand spatial structure indices have not been fully elucidated.

Species diversity has long been a central theme in ecology, with higher diversity generally assumed to enhance ecosystem resistance to disturbance and overall stability (Tilman, 1999). Recent research has highlighted the close relationship between stand spatial structure and understory vegetation diversity. While understory vegetation is predominantly composed of shrubs and herbaceous species, saplings are of particular importance because they underpin stand regeneration. Much of the recent work on saplings has emphasized the influence of external factors such as disturbance and site conditions (Danyagri et al., 2019). Stand structural variables, including plot area, stand age, and canopy gaps, also play significant roles (Wang et al., 2017). Despite this progress, whether stand spatial structure consistently regulates species diversity remains unclear. Evidence indicates that stand spatial structure has a strong influence on sapling regeneration (Long et al., 2021), yet few studies have explicitly examined how the spatial arrangement of neighboring trees shapes sapling diversity. Incorporating stand spatial structure based on nearest-neighbor relationships into analyses of sapling dynamics may provide deeper insights into competitive interactions and the mechanisms underlying diversity maintenance.

The tropical karst seasonal rainforest in the northern tropics represents a unique ecosystem within global karst landscapes. As a characteristic forest type at the northern margin of the tropical karst region in China, it is distinguished by its complex ecological processes and high biodiversity. Although numerous studies have been conducted in this region, the spatial structural attributes of stands and their impact on sapling diversity remain insufficiently explored. This study addresses this gap by investigating the dynamics of stand spatial structure, identifying the key factors that shape it, and evaluating its effects on sapling diversity. Specifically, we aim to answer the following questions: (1) What are the dynamic characteristics of stand spatial structure? (2) What are the main factors influencing stand spatial structure? (3) How does stand spatial structure affect sapling growth?

## 2 Materials and methods

### 2.1 Study site

The study was conducted in the Nonggang National Natural Reserve, located in the southwest of Guangxi Zhuang Autonomous Region, southern China (22°13′56”-22°33′09”N, 106°42′28”-107°04′54”E). The reserve spans the eastern part of Longzhou County and the northern part of Ningming County, covering an area of 10,080 hectares. The region is characterized by a distinct seasonal climate with pronounced wet and dry periods. Topographically, it exhibits a typical karst peak-cluster depression landscape, with relative elevation differences of 250–300 m between mountaintops and depressions, producing a highly heterogeneous terrain. The mean annual temperature is approximately 22°C, and the annual precipitation ranges from 1,200 to 1,500 mm, most of which occurs from May to September. These conditions support the high biodiversity and distinctive ecosystem of the Nonggang Nature Reserve. The reserve protects a rare, large-scale, and relatively intact karst seasonal rainforest, which is of global conservation significance. Representative tree species include *Excentrodendron tonkinensis*, *Garcinia paucinervis* and *Cephalomappa sinensis*.

### 2.2 Plot establishment

The 15-hectare monitoring plot of the northern tropical karst seasonal rainforest is located in the Nongmuhuang area of the Nonggang section of the Nonggang Nature Reserve. The plot features a rectangular layout, extending 500 m east-west and 300 m north-south. It encompasses both a small karst peak and a relatively intact depression. Elevation within the plot ranges from 180 to 370 m, with a mean of approximately 260 m. Slope conditions vary markedly, from gentle inclines of 3.7° to steep slopes of 78.9°, with an average slope of 41.7°. The plot was established in 2011, followed by a vegetation survey. Re-examinations have since been conducted at five-year intervals, with the second completed in 2021 (Guo et al., 2017b). In this study, we analyzed stand spatial structure parameters using individual tree data with diameter at breast height (DBH) ≥ 5 cm collected in 2011 and 2021. Saplings with DBH < 5 cm were also included to assess diversity and regeneration dynamics.

### 2.3 Data analyses

#### 2.3.1 Stand spatial structure

Three parameters, namely the uniform angle index, dominance, and mingling (Li et al., 2012) (**Table 1**), were selected to analyze the stand spatial structure of the northern tropical karst seasonal rainforest from 2011 to 2021. Spatial structure was characterized using zero, univariate, bivariate, and trivariate distribution of these parameters. The zero distribution represents the scenario in which each of the three spatial structural parameters take its mean value. The univariate distribution describes the frequency of five possible value levels for any single structural parameter. The bivariate distribution was derived by systematically pairing structural parameters across their ordinal levels, generating 25 distinct relative frequency distributions. The trivariate distribution involves the cross-classification of five value levels for two parameters (X and Y) with the five value levels of a third parameter (Z), yielding 5×5×5=125 spatial structural combinations and their corresponding relative frequency distributions.

**Table 1:** Stand spatial structure parameters and their formulas.

Spatial structure parameters	Equation at individual level	Equation at stand level
Uniform angle index	${\text{W}}_{\text{i}}=\frac{1}{4}\sum_{\text{j}=1}^{4}{\text{Z}}_{\text{ij}}$	$\bar{\text{W}}=\frac{1}{\text{N}}\sum_{\text{i}=1}^{\text{N}}{\text{W}}_{i}$
Dominance	${\text{U}}_{\text{i}}=\frac{1}{4}\sum_{\text{j}=1}^{4}{\text{K}}_{\text{ij}}$	$\bar{\text{U}}=\frac{1}{\text{N}}\sum_{\text{i}=1}^{\text{N}}{\text{U}}_{i}$
Mingling	${\text{M}}_{\text{i}}=\frac{1}{4}\sum_{\text{j}=1}^{4}{\text{V}}_{\text{ij}}$	$\bar{\text{M}}=\frac{1}{\text{N}}\sum_{\text{i}=1}^{\text{N}}{\text{M}}_{i}$

i is the reference tree, j is an adjacent tree, and N is the number of trees in the plot after eliminating edge effects.

Relative frequency was calculated by dividing the number of individuals in each value or value combination by the total number of individuals. The uniform angle index, dominance index, and mingling index were each classified into five distinct value levels: 0, 0.25, 0.5, 0.75, and 1. For the uniform angle index, these values correspond to very uniform, uniform, random, irregular, and very irregular distributions, respectively. For the dominance index, the five values represent predominant, subdominant, intermediate, disadvantaged, and absolutely disadvantaged. For the mingling index, the five values correspond to zero mingling, weak mingling, medium mingling, high mingling, and complete mingling. To minimize edge effects, a 5 m buffer zone was applied to the plot.

To quantify individual dynamics, live trees, recruited trees, and dead trees were identified between 2011 to 2021. Sankey diagrams were then employed to visualize transitions among these categories, and the proportions of change in the three processes were calculated to reveal dynamic shifts in stand spatial structure at the individual tree level. Tree recruitment was defined as individuals that either germinated after 2011 or had DBH < 5 cm in 2011 but grew to DBH ≥ 5 cm by 2021. Tree survival was defined as individuals that were alive in both 2011 and 2021. Tree mortality referred to individuals with DBH ≥ 5 cm in 2011 that had died by 2021.

The values of surviving trees exhibited three possible trends: “ascending”, “stable” and “descending” (Zhou et al., 2022). For example, an increase in value from 0 to 0.25 was defined as “ascending one-step (Flow+1)”, whereas a decrease from 1 to 0 was defined as “four-step descending (Flow-4)”. Cases in which the value remained unchanged were defined as “stable”. In total, nine possible value-change scenarios were recognized for surviving trees. By comparison, five value-change scenarios were applied to tree recruitment and mortality processes.

We used the *forestSAS* (https://CRAN.R-project.org/package=forestSAS) package in R 4.3.1 (https://www.r-project.org) to analyze the stand spatial structure, and the *ggalluvial* package to draw Sankey diagrams.

#### 2.3.2 Relationship between spatial structure parameters and environmental factors

Spatial structural parameters and environmental factors were calculated for each 20 m×20 m sample plot. The environmental factors primarily include topographic factors such as elevation, aspect, slope, convexity (Harms et al., 2001; Valencia et al., 2004), topographic wetness, elevation above channel, and rock-bareness rate (RBR). Calculation methods for these indices followed Punchi-Manage et al. (2013) and Kanagaraj et al. (2011). Aspect was transformed using the following formula:

(1) TRASP={1−cos[(π/180)(aspect−30)]}/2

where TRASP denotes the transformed aspect index, and aspect is the slope direction angle (°). This transformation normalizes TRASP values between 0 and 1. Higher TRASP value indicate drier and hotter habitats: a TRASP value of 0 corresponds to the north-northeast direction, whereas a TRASP value of 1 corresponds to the south-southwest direction.

The factors included maximum diameter at breast height, the sum of the area at breast height, the number of individual plants (Ind), and mean diameter at breast height. When calculating structural parameters for each 20 m×20 m plot, a 2 m buffer zone was applied.

To determine whether the stand spatial structure exhibited spatial autocorrelation, we performed a global spatial autocorrelation analysis using the following calculation formula:

(2) $$\text{I}=\frac{\text{n}}{{\text{S}}_{0}}\cdot \frac{\sum_{\text{i}=1}^{\text{n}}\sum_{\text{j}=1}^{\text{n}}{\text{w}}_{\text{ij}}({\text{x}}_{i}-\bar{\text{x}})({\text{x}}_{j}-\bar{\text{x}})}{{\sum_{\text{i}=1}^{\text{n}}\left({\text{x}}_{i}-\bar{\text{x}}\right)}^{2}}$$

where S_0_ is the sum of all elements in the spatial weights matrix; n is the number of spatial units, x_i_ and x_j_ are the attribute values of unit i and unit j respectively, x̄ is the mean of all attribute values, and w_ij_ is the spatial weight between units i and unit j (de Campos et al., 2024).

Based on the spatial dependence of stand spatial structure data and the objectives of this study, both the spatial lag model (SLM) and spatial error model (SEM) were constructed to systematically analyze spatial autocorrelation and identify the key driving factors of stand spatial structure (Wang et al., 2019). The SLM is expressed as:

(3) Y=ρWY+Xβ+ϵ

The SEM is expressed as:

(4) Y=Xβ+λWμ+ϵ

where Y is the dependent variable matrix, X is the explanatory variable matrix, W is the spatial weight matrix, β is the vector of regression coefficients, and ϵ is the model residual. The terms ρ, λ, and γ represent spatial autoregressive coefficients, and *μ* denotes the spatially correlated residual.

In the global spatial autocorrelation analysis, the mingling index and uniform angle index exhibited significant positive spatial correlations, whereas the dominance index showed no significant spatial autocorrelation. Furthermore, results from both the SLM and SEM yielded non-significant, indicating that neither the spatial structure of the stand nor its influencing factors showed significant spatial autocorrelation (**Table 2**).

**Table 2:** Spatial autocorrelation of stand spatial structure in northern tropical karst seasonal rainforests.

Spatial structure parameter	Moran’s I	P (Moran’s I)	Moran’s I (SEM)	P (SEM)	Moran’s I (SLM)	P (SLM)
Uniform angle index	0.21	0.00	-0.00	0.50	0.00	0.48
Dominance	0.01	0.35	0.00	0.48	0.01	0.40
Mingling	0.41	2.2e-16	-0.02	0.65	-0.02	0.71

P < 0.01 indicates statistical significance; P < 0.001 indicates high statistical significance.

Then the impact of environmental factors on the spatial structure of the stand was analyzed using generalized additive models (GAM). The GAMs are well suited for fitting non-linear relationship between the response variable and multiple explanatory variables. In this study, the smoothing function was specified as the default thin-plate spline with degrees of freedom set to 8. Prior to model fitting, pairwise correlation coefficients were calculated among the explanatory variables, and a threshold of |r|>0.7 was used to identify potential multicollinearity. Variance inflation factors (VIF) were then calculated, with values < 10 indicating no substantial collinearity. Model reliability was evaluated using the deviance explained, while the significance of explanatory variables was assessed using F-tests. To further evaluate the relative importance of explanatory variables, the fitted GAM was decomposed using the *gam.hp* package to obtain contribution rates (Lai et al., 2024). The expression for the GAM is:

(5) $$\text{G}[\text{E}(\text{Y})]={\text{β}}_{0}+{\text{f}}_{1}({\text{x}}_{1})+\ldots +{\text{f}}_{\text{n}}({\text{x}}_{\text{n}})$$

where E(Y) is the expected value of the response variable, G[] is the link function determined by the distribution of the response, β_0_ is the intercept, and f_1_, …, f_n_ are smooth functions of the n explanatory variables, usually estimated using smoothing splines (Wood, 2006).

#### 2.3.3 Relationship between spatial structure parameters and saplings diversity

In this study, four diversity indices were used to characterize sapling diversity: the Shannon-Wiener index (H), the Simpson index (D) (Simpson, 1949), the Pielou evenness index (J) (Pielou, 1966), and the Margalef richness index (F) (Pielou, 1966). The effects of stand spatial structure on sapling diversity were analyzed using GAMs, following the methodology described above. The indices were calculated as follows:

Shannon-Wiener index:

(6) $$\text{H}=-\sum_{\text{i}=1}^{\text{S}}{\text{P}}_{\text{i}}{\text{logP}}_{\text{i}}$$

Simpson index:

(7) $$D=1-\sum_{i=1}^{S}P_{i}^{2}$$

Pielou index:

(8) J=H/logS

Margalef index:

(9) F=(S−1)/lnN

where S is the number of species, P_i_ is the proportion of individuals of species i relative to the total number of individuals, and N is the total number of individuals of all species.

## 3 Results

### 3.1 Characteristics of stand spatial structure

#### 3.1.1 Zero and Univariate distribution of stand spatial structure

**Figure 1:**
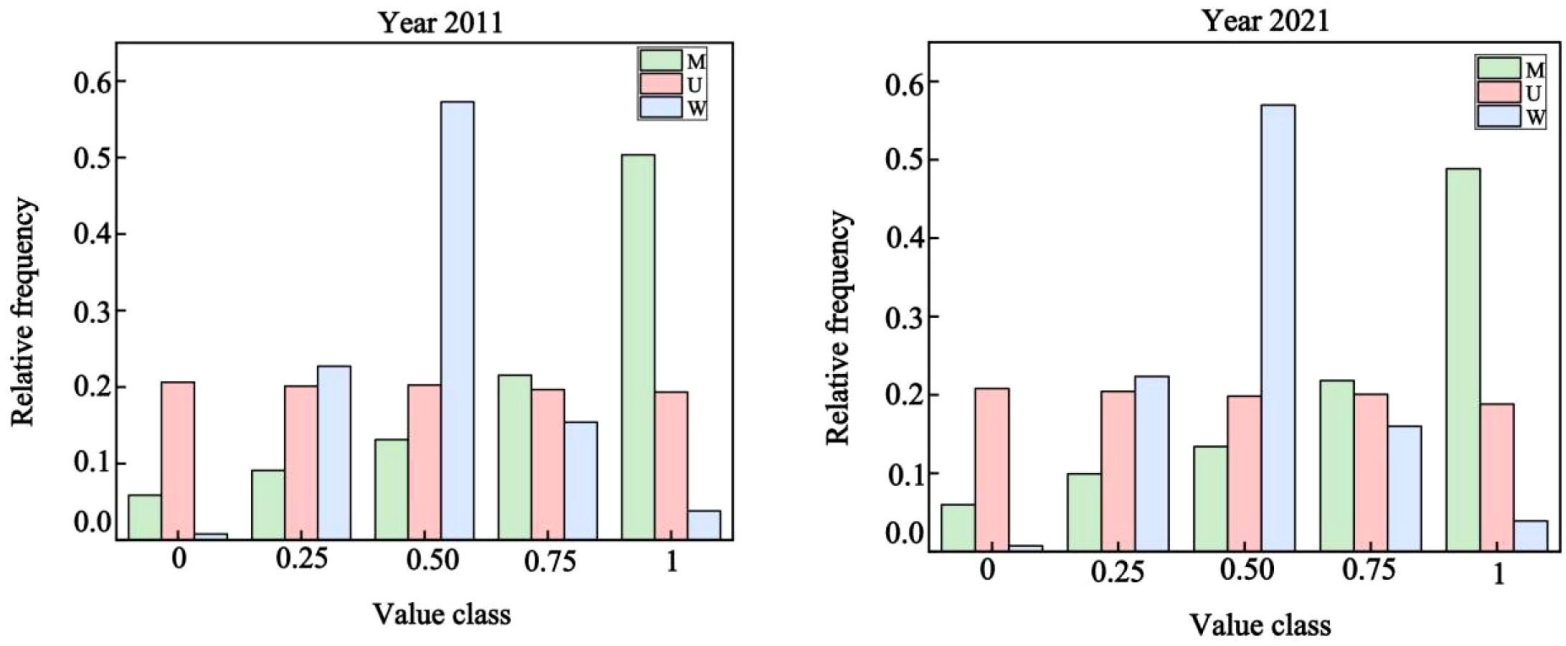
Univariate distributions of uniform angle index, dominance index, and mingling index in the tropical karst seasonal rainforest in 2011 and 2021.

The distributions of spatial structure parameters in 2011 and 2021 exhibited consistent patterns. For the uniform angle index, relative frequency increased initially and then decreased with higher levels, approximating a normal distribution. Across the sample plots, values of the uniform angle index generally fell within the range of random distribution, with more than half of the trees randomly dispersed throughout the study area. In contrast, very uniformly distributed trees were the least frequent. For the mingling index, low mingling levels consistently occurred at low frequencies, whereas relative frequency increased with higher mingling levels. In both years, the stand exhibited a strongly mixed state, indicating high species heterogeneity. The frequency distribution of the five dominance levels was relatively balanced, with overall dominance reflecting an intermediate state. This pattern suggests a neutral competitive balance among trees and stable equilibrium in individual competition dynamics (**Table 3**; **Figure 1**).

**Table 3:** Average values of spatial structure parameters in the tropical karst seasonal rainforest in 2011 and 2021.

Year	Uniform angle index	Dominance	Mingling
2011	0.50	0.49	0.75
2021	0.50	0.49	0.75

#### 3.1.2 Bivariate distribution of stand spatial structure

**Figure 2:**
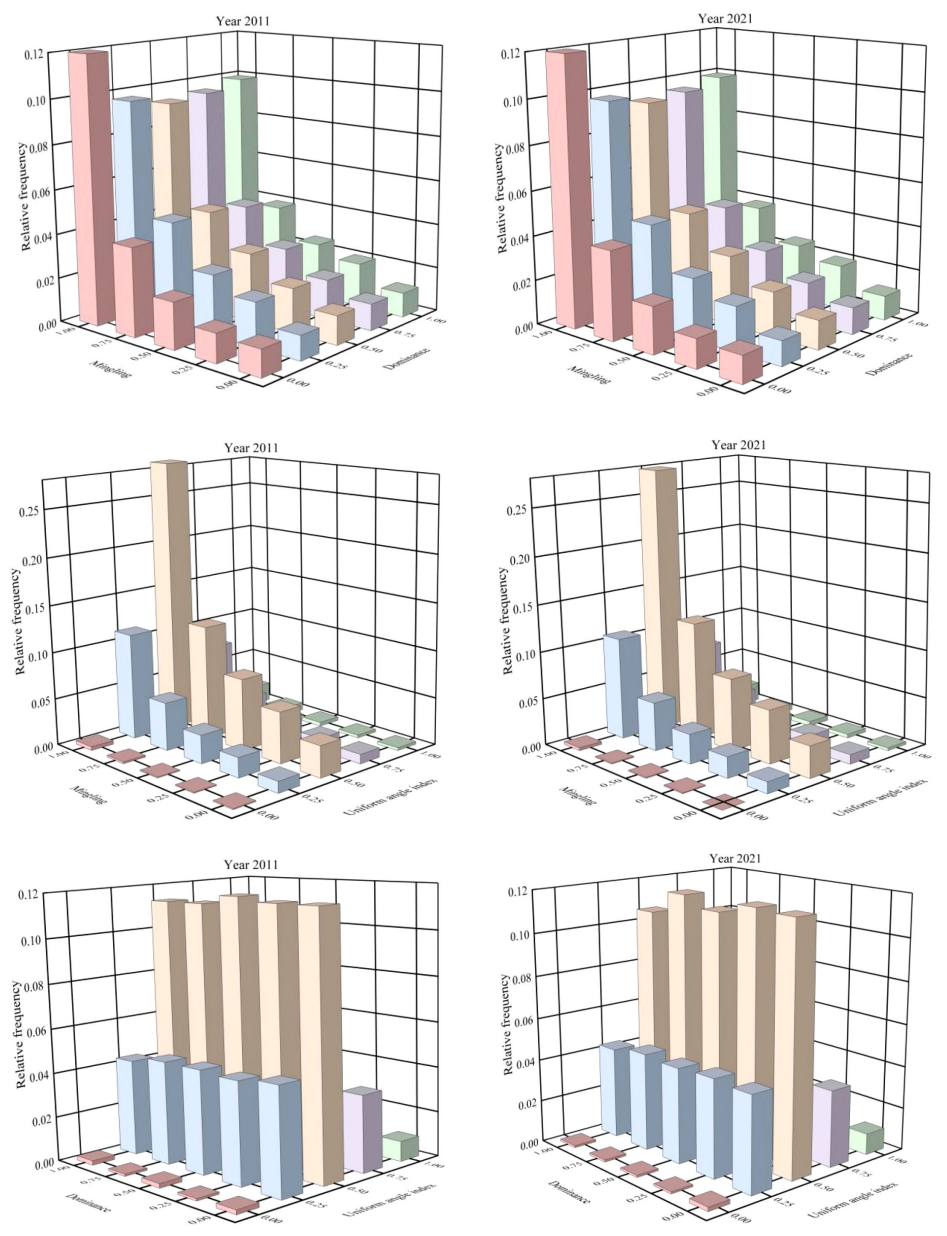
Bivariate distributions of uniform angle index, dominance index, and mingling index in the tropical karst seasonal rainforest in 2011 and 2021.

The spatial structure distribution of the stand exhibited a high degree of similarity between 2011 and 2021. In both years, the relative frequency within each dominance class consistently increased with higher levels of mingling, reaching its peak under complete mingling conditions. The maximum frequency occurred when the dominance was 0 and the mingling degree reached 1.00. Under zero mingling, the relative frequencies of trees across different competitive states were nearly identical, whereas higher mingling levels revealed more pronounced variations in frequency distribution among dominance classes (**Figure 2**).

For the uniform angle index, frequencies generally increased with greater mingling intensity, except under conditions of absolute uniformity. Within each mingling level, uniform angle index frequencies followed a normal distribution. The highest frequencies occurred under strong mingling combined with random spatial distribution. In contrast, frequencies were close to zero when absolute uniformity was paired with zero mingling, indicating that non-mingled trees were exceedingly rare in highly uniform stands (**Figure 2**).

Notably, when the uniform angle index was held constant, relative frequencies showed only minor variation across dominance classes. In contrast, within the same dominance level, uniform angle index frequencies exhibited a symmetrical decline from a central peak at random distribution, decreasing toward both clumped and uniform extremes. Randomly distributed trees constituted the majority of the stand, whereas absolutely uniform distribution was the rarest spatial pattern, underscoring its structural scarcity in the forest (**Figure 2**).

#### 3.1.3 Trivariate distribution of stand spatial structure

**Figure 3:**
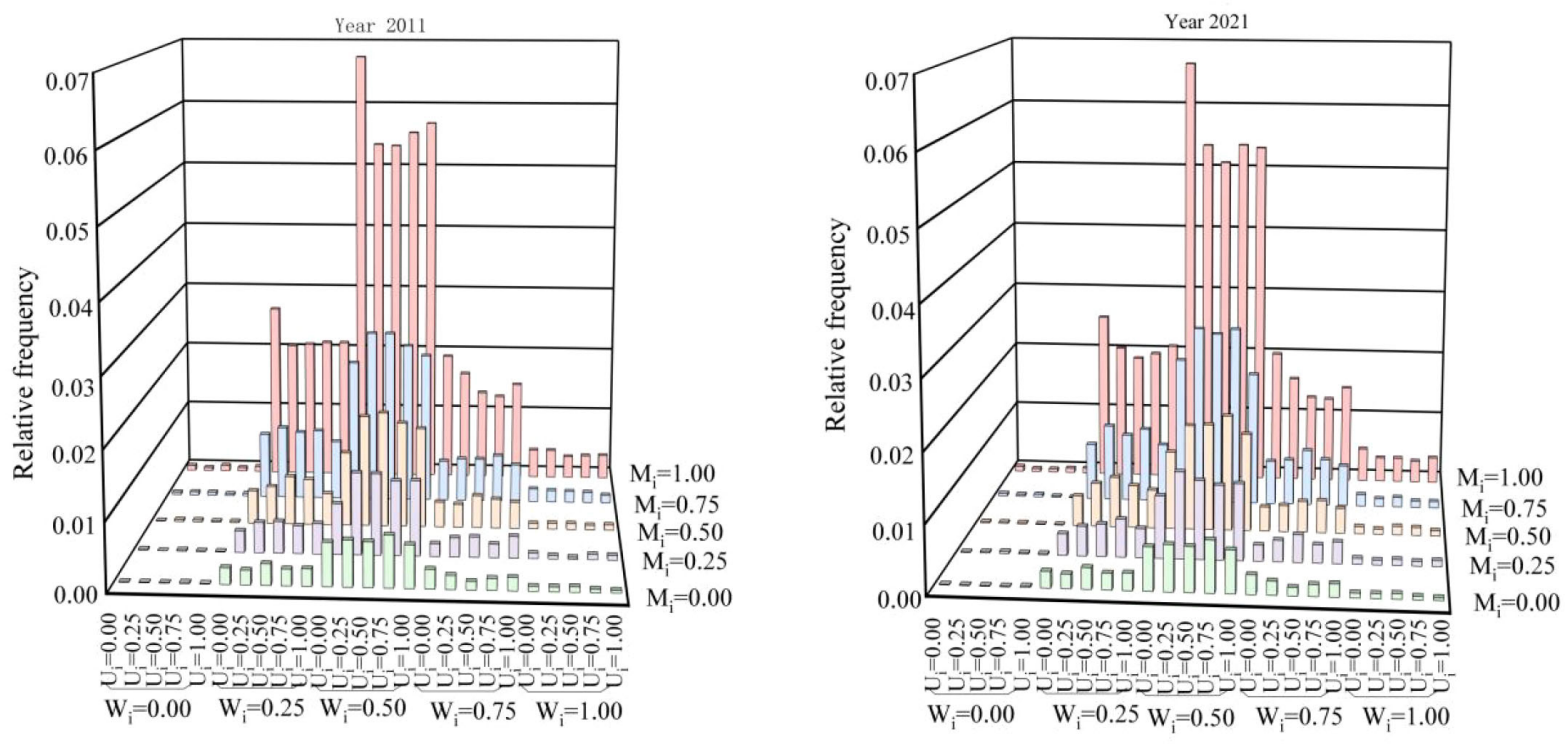
Trivariate distribution of uniform angle index, dominance index, and mingling index in the tropical karst seasonal rainforest in 2011 and 2021.

In both 2011 and 2021, the trivariate distribution of spatial structure showed that most trees within the same mingling and dominance classes exhibited random spatial patterns. Under identical mingling and uniform angle index conditions, relative frequency did not display a consistent trend with changes in dominance. However, at the same dominance and uniform angle index, frequencies increased gradually with rising mingling intensity. In both years, the highest relative frequencies occurred under random distribution combined with intermediate dominance and very high mingling intensity (**Figure 3**).

### 3.2 The individual level variation in stand spatial structure

**Figure 4:**
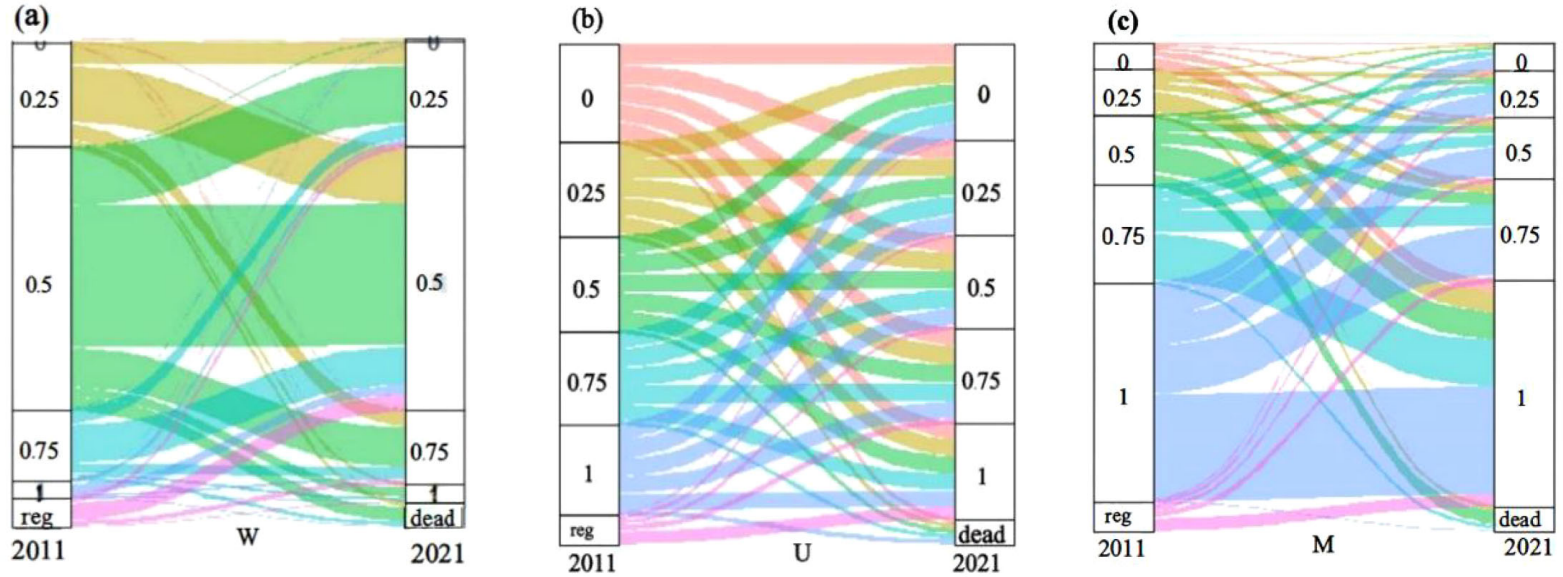
Dynamic characteristics of spatial structure of uniform angle index **(a)**, dominance index **(b)**, and mingling index **(c)** in the northern tropical karst seasonal rainforest. reg, Trees with DBH≥5 cm that regeneration ingrowth in 2021; dead, Trees that were alive with DBH≥5 cm in 2011 but died by 2021.

From 2011 to 2021, the spatial structure parameters of living trees in the northern tropical karst seasonal rainforest exhibited very few four-step changes, while three-step and two-step changes were also relatively uncommon. In contrast, one-step changes and unchanged states predominated. Among the unchanged parameters, the stability of uniform angle index and mingling index was significantly higher compared to the dominance index (**Table 4**; **Figure 4**).

**Table 4:** Proportional statistics of flow changes in living trees in the seasonal rainforest of the northern tropical karst.

Types	Uniform angle index (%)	Dominance (%)	Mingling (%)
Flow+4	0.03	3.74	3.01
Flow+3	0.94	7.96	5.87
Flow+2	6.52	11.95	8.99
Flow+1	22.72	15.84	15.23
Flow0	40.13	20.13	32.81
Flow-1	22.80	15.99	15.43
Flow-2	5.79	12.27	9.40
Flow-3	1.04	8.22	6.27
Flow-4	0.04	3.91	3.00

Flow0: Structure parameter value level: 0→0, 0.25→0.25, 0.5→0.5, 0.75→0.75, 1→1; Flow+1: 0→0.25, 0.25→0.5, 0.5→0.75, 0.75→1; Flow+2: 0→0.25, 0.25→0.75, 0.5→1; Flow+3: 0→0.75, 0.25→1; Flow+4: 0→1. The ascending direction is opposite.

During tree mortality, the uniform angle index and mingling showed the lowest proportions in the Dead-0, whereas the dominance had its lowest proportion in Dead-3. The highest proportions occurred in Dead-2 for the uniform angle index, in Dead-4 for the mingling index, and in Dead-0 for the dominance index. Notably, dominance also maintained relatively high proportions in Dead-1 and Dead-4, in addition to Dead-0 (**Table 5**; **Figure 4**).

**Table 5:** Proportional statistics of flow changes with the mortality process in the seasonal rainforest of the northern tropical karst.

Types	Uniform angle index (%)	Dominance (%)	Mingling (%)
Dead0	0.63	20.92	3.77
Dead-1	21.02	20.61	6.80
Dead-2	57.43	19.46	14.23
Dead-3	16.21	18.41	21.76
Dead-4	4.71	20.61	53.45

Dead0: Structure parameter value level 0→0; Dead-1: 0.25→0; Dead-2: 0.5→0; Dead-3: 0.75→0; Dead-4: 1→0.

In the recruitment process, the uniform angle index showed the highest rate of change at Reg+2, followed by Reg+1 and Reg+3, while remaining most stable at Reg+0. The dominance index peaked at Reg+3, with Reg+4 as the secondary peak, and reached its lowest proportion at Reg+2. The mingling index displayed the greatest variability at Reg+4, followed by Reg+3, whereas Reg+0 represented the most stable state (**Table 6**; **Figure 4**).

**Table 6:** Proportional statistics of flow changes with the recruitment process in the seasonal rainforest of the northern tropical karst.

Types	Uniform angle index (%)	Dominance (%)	Mingling (%)
Reg0	0.53	19.17	6.74
Reg+1	18.72	17.57	12.69
Reg+2	59.63	19.08	15.17
Reg+3	16.50	22.36	21.15
Reg+4	4.61	21.83	43.92

Reg0: Structure parameter value level 0→0; Reg+1: 0→0.25; Reg+2: 0→0.5; Reg+3: 0→0.75; Reg+4: 0→1.

### 3.3 Drivers of stand spatial structure

**Figure 5:**
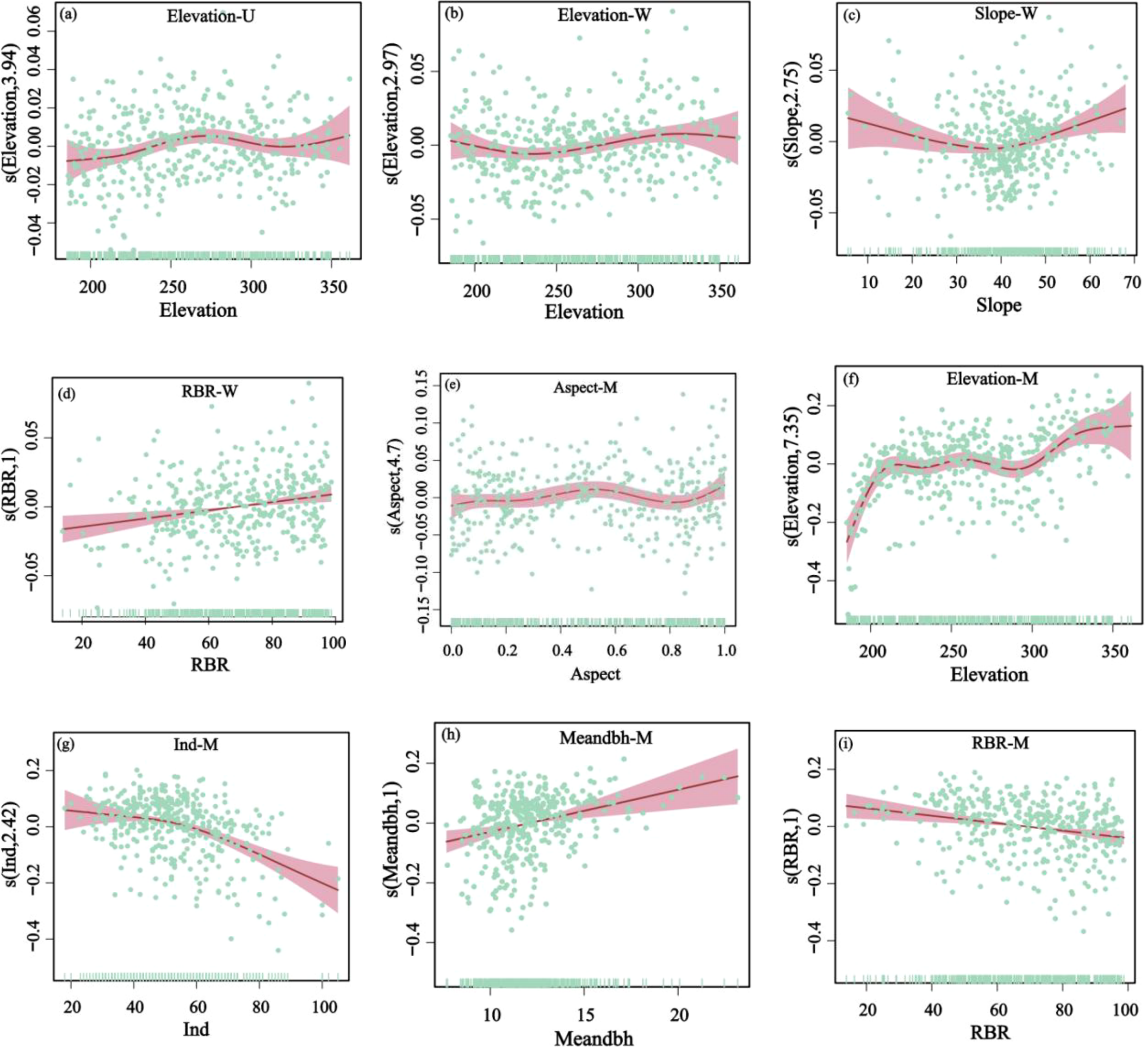
Relationship between the uniform angle index (W), dominance index (U), and mingling index (M) of the tropical karst seasonal rainforest stand andenvironmental factors. Relationships between the elevation with the dominance index **(a)**, uniform angle index **(b)**, and mingling index **(f)**; relationship between the slope with the uniform angle index **(c)**; relationships between the rock-bareness rate (RBR) with the uniform angle index **(d)** and mingling index **(i)**; relationship between the aspect with the mingling angle index **(e)**; relationship between the number of individualstrees for each 20 m × 20 m sample plot (Ind) with the mingling angle index **(g)**; relationship between the meandbh with the mingling angle index **(h)**.

The GAMs accounted explained approximately 9.73% of the variation in dominance with respect to influencing factors. A nonlinear relationship was detected between elevation and dominance: dominance increased with elevation at lower ranges, declined thereafter, and reached a secondary peaked at around 250 m. Elevation played the primary role in driving dominance. Among explanatory variables, topographic factors accounted for 95.83% of the explained variation, whereas biotic factors contributed only 4.17% (**Tables 7**, **8**; **Figure 5a**).

**Table 7:** GAM fitting and hierarchical partitioning of spatial structure parameters and influencing factors.

Spatial structure parameters	Environmental factors	P	I.perc (%)	Deviance explained (%)
Mingling	Meandbh	0.001***	8.55	47
	Elevation	<2e-16***	31.35	
	Aspect	0.021*	5.33	
	RBR	0.001***	4.26	
	Ind	<2e-16***	24.13	
Uniform angle index	Elevation	0.037*	16.63	29.20
	Slope	0.003**	22.53	
	RBR	0.001***	20.04	
Dominance	Elevation	0.038*	64.99	8.46

***P<0.001; **0.001<P<0.01; *0.01<P<0.05.

**Table 8:** Hierarchical partitioning of explanatory variables by biotic and topographical factors groups.

Spatial structure parameters	Influencing factors	I.perc (%)
Mingling	Biotic	40.81
	Topographic	59.19
Uniform angle index	Biotic	24.01
	Topographic	75.99
Dominance	Biotic	4.17
	Topographic	95.83

Results from the GAMs indicated that the uniform angle index and its influencing factors together explained 30.8% of the total deviance. A significant linear relationship was detected between the rock-bareness rate and the uniform angle index: as the rock-bareness rate increased, the uniform angle index also increased significantly (**Figure 5d**). In contrast, the relationships of the uniform angle index with elevation and slope exhibited significant nonlinear trends (**Figures 5b, c**). With increasing elevation, the uniform angle index followed a “decrease–increase–decrease” pattern. As slope increased, the uniform angle index showed a “decrease–increase” pattern, reaching its minimum value at 40°. Hierarchical partitioning of explanatory variables showed that topographic factors accounted for a greater proportion of explained variation than biotic factors (**Tables 7**, **8**).

Results from the GAMs indicated that mingling was significantly influenced by mean diameter at breast height, elevation, aspect, rock-bareness rate, and number of individual plants. Together these variables explained 46.6% of the total variation. Rock-bareness rate and mean diameter at breast height showed positive linear correlations with mingling (**Figures 5h, i**), whereas the relationships of mingling with aspect, elevation, and number of individual plants were nonlinear. Mingling declined gradually with changes in aspect, reaching its lowest value when the aspect approached 1 (**Figure 5e**). With increasing elevation, mingling followed a complex multi-peak trend, peaking at the highest elevation (**Figure 5f**). In contrast, mingling decreased steadily as number of individual plants increased (**Figure 5g**). Hierarchical partitioning of explanatory variables showed that topographic factors accounted for a larger proportion of the explained variation than biotic factors (**Tables 7**, **8**).

### 3.4 The impact of stand spatial structure on the sapling diversity

**Figure 6:**
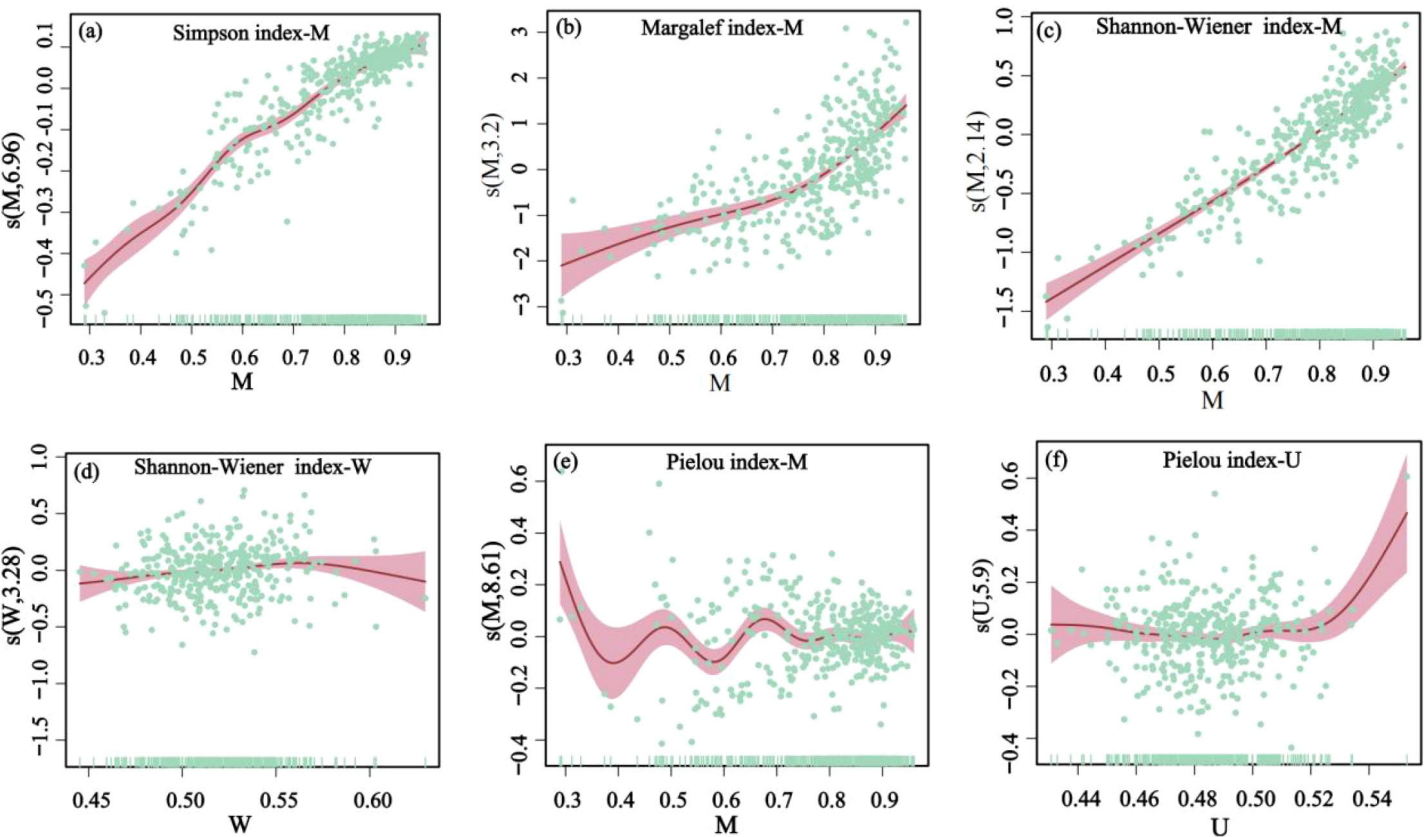
The GAM fits of the relationships between the uniform angle index (W), dominance index (U), and mingling index (M) in relation to tropical karst seasonal rainforest stands saplings diversity index. Relationships between the mingling index (M) with the Simpson index **(a)**, Margalef index **(b)**, Shannon-Wiener index **(c)**, and Pielou index **(e)**; relationship between the uniform angle index (W) with the Shannon-Wiener index **(d)**; relationship between the dominance index (U) with the Pielou index **(f)**.

Based on the GAMs results, mingling was significantly correlated with all four diversity indices. As the mingling increased, the Shannon-Wiener index (**Figure 6c**), Simpson index (**Figure 6a**), and Margalef index (**Figure 6b**) exhibited upward trends, while the Pielou evenness index (**Figure 6e**) showed a multi-peaked fluctuation pattern. The uniform angle index showed a significant nonlinear relationship only with the Shannon–Wiener index (**Figure 6d**), which increased initially and then declined; however, its contribution rate was only 0.26%. In addition, the dominance index exhibited a significant linear relationship with the Pielou index (**Figure 6f**): as dominance increased, the Pielou index initially remained stable and then gradually rose, with a contribution rate of 40.54% (**Table 9**). Nevertheless, this relationship was no longer significant after accounting for extreme values.

**Table 9:** The GAM fitting results of sapling diversity and spatial structure parameters in tropical karst seasonal rainforests.

Sapling diversity index	Spatial structure parameters	P	I.perc (%)	Deviance explained (%)
Shannon-Wiener index	Mingling	<2e-16***	99.68	76.60
	Uniform angle index	0.026*	0.26	
Simpson index	Mingling	<2e-16***	99.70	81.80
Pielou index	Mingling	0.001***	54.88	14.40
	Dominance	0.005**	40.54	
Margalef index	Mingling	<2e-16***	99.69	46.50

***P<0.001; **0.001<P<0.01; *0.01<P<0.05.

## 4 Discussion

### 4.1 The dynamic changes of spatial structure at the stand level

As an important part of forest structure, changes in stand spatial structure are a key driver of community succession (Yang et al., 2019). Our results showed that, at the stand level, only minor changes occurred in stand spatial structure between 2011 to 2021. Instances of conspecific clustering were relatively rare, and most trees were spatially associated with heterospecific neighbors. Moreover, competitive relationships among trees appeared relatively balanced, with no clear advantages or disadvantages in competition. In the univariate distribution, randomly distributed individuals accounted for the largest proportion. In the trivariate distribution, trees with the same mingling and dominance levels were randomly distributed, and the overall stand spatial structure index remained stable across the decade. Taken together, these characteristics suggest that the northern tropical karst seasonal rainforest has developed toward a climax community stage, characterized by high species heterogeneity and largely independent interactions among individual trees.

In climax communities, stands typically exhibit random distribution under equilibrium conditions. This occurs because, after long-term development and succession, once a stand reaches stability and maturity, previously clustered trees die at random due to various natural causes, gradually giving rise to a random distribution pattern. Our results indicated that, overall, the stand followed a random distribution, although exceptions were observed under different environmental conditions. For example, microenvironment variation, site-specific regulation, and natural disturbances, such as trees withering and falling due to storms, rain, and snow, can alter tree regeneration dynamics and shift distribution patterns. Additionally, species invasion may disrupt stand structure and modify distributional states. The “random structural framework-stability” hypothesis suggests that randomly distributed trees constitute the primary structural patter of natural forests, regardless of geographical distribution, stand type, species composition, or distributional form (Hui et al., 2021). Our findings support this hypothesis and align with previous results (Zhang and Hui, 2021).

### 4.2 Individual level spatial structural dynamics of stand

During individual tree growth, the distribution pattern demonstrates the highest stability, followed by mingling, whereas size differentiation exhibits the lowest stability. This observation is consistent with Zhou et al. (2022). Instability in size differentiation occurs when the reorganization of forest structural units is triggered by the death or recruitment of neighboring trees, which alters the size relationships between reference trees and their nearest neighbors. Such instability may also result from inherent shifts in growth dynamics between reference trees and adjacent individuals. In contrast, changes in the uniform angle index and mingling were primarily driven by spatial rearrangements caused by neighbor mortality or recruitment, which modified the relative positions and species composition surrounding reference trees. Tree survival was largely governed by individual growth rates, which were more sensitive to habitat constraints. Consequently, compared with distribution and segregation patterns, size differentiation was more likely to change, reflecting the distinct dynamic behaviors of different forest structural indices.

Tree recruitment and mortality play dominant roles in reshaping stand spatial structure and are critical drivers of forest dynamics (Kanagaraj et al., 2011). Our results indicated that mortality increases the proportion of reference trees exhibiting absolute uniformity in distribution, inferior size differentiation, and zero mingling. Conversely, recruitment elevated the proportion of reference trees with random distribution, inferior size differentiation, and high mingling. Relative to the overall stand spatial structure, mortality accentuated distributional differences, while recruitment enhanced mingling. These two processes therefore exert opposing influences on stand spatial structure. Furthermore, as stands develop, both mortality and recruitment gradually shift trees into more disadvantaged states.

Tree mortality arises from two primary causes: intrinsic biotic traits and the combined effects of environmental factors. These include competitive interactions, both intra- and interspecific, within a given spatial range. Competition-induced mortality reduces species segregation and weakens tree aggregation over time, thereby shifting distribution patterns toward greater uniformity. When dominant trees die as a result of competition, previously suppressed individuals may benefit from released resources and persist. Thus, competition drives divergent trajectories in tree distribution, size differentiation, and segregation. Meanwhile, recruitment increases tree density, promotes clustering, elevates the likelihood of heterospecific neighbors, and shifts initially dominant trees into more disadvantageous states. Taken together, recruitment and mortality are central processes that regulate stand dynamics and play a fundamental role in maintaining long-term forest stability and equilibrium.

### 4.3 Factors influencing stand spatial structure

#### 4.3.1 Elevation

Elevation emerged as a key factor significantly influencing three spatial structure parameters. Overall, dominance increased with elevation, reaching its highest values at mid-slopes and hilltops. This pattern suggests that species size differentiation gradually decreased with elevation: trees in depressions exhibited greater dominance, whereas size differentiation was weaker at mid-slope and summit positions. A plausible explanation is that intense competition for water and light in rocky, high-elevation areas promotes the concentration of large-diameter individuals in depressions, while smaller trees persist at higher elevations, thereby reducing overall dominance. The pronounced variation in dominance across elevation gradients confirms elevation as a primary driver of size differentiation.

Elevation significantly influences plant distribution and growth both directly and indirectly by regulating key a factors such as light, temperature, water availability, and soil conditions (Zhao et al., 2005). The uniform angle index declined gradually with elevation before rising again, reaching its lowest values at 200–250 m. This pattern indicates more dispersed tree distributions in depressions and mid-slopes, but increasingly clustered patterns at hilltops. Elevational shifts in species richness are accompanied by intensified interspecific competition, self-thinning, and reduced intraspecific clustering. At higher elevations, increased rock exposure amplifies competition for light and heat, forcing trees into clustered distributions under harsh habitat conditions. Conversely, lower-elevation depressions and mid-slopes offer more favorable growth conditions, promoting dispersed tree distributions.

Elevation also strongly influences species segregation. The mingling index increased continuously in depressions, remained relatively stable at mid-slopes, and rose again at hilltops. Spatial segregation was lowest in depressions, moderate at mid-slopes, and highest at hilltop and bealocks. Areas with high segregation were characterized by strong species heterogeneity, elevated tree diversity, and more stable forest structures, often accompanied by a higher proportion of dominant species. This pattern likely reflects optimal light and hydrothermal conditions at higher elevations, which maximize resource availability, coupled with intensified intraspecific competition and self-thinning. However, Lv et al. (2023) reported that mingling increased with elevation while the dominance index and uniform angle index were unaffected, a discrepancy potentially attributable to karst habitat heterogeneity, where complex terrain alters structural parameter responses to elevation.

#### 4.3.2 Slope and aspect

Slope and aspect significantly influence solar radiation, temperature, and precipitation distribution, thereby shaping the spatial arrangement and distribution of trees. Variations in slope gradient affect soil moisture content, which in turn regulates tree distribution. Gentle slopes exhibit superior soil water retention and nutrient preservation compared to steep slopes. As slope increased, the uniform angle index declined, reaching its minimum at approximately 40°, and then rose again beyond this threshold. Slope was identified as the primary driver of these changes in the uniform angle index. When the slope approached 40°, tree distribution tended tree distribution tended toward randomness, coinciding with optimal soil water-holding capacity and nutrient availability. In contrast, other slope ranges exhibited weaker soil conditions, resulting in more clustered tree distributions. Lin et al. (2021) reported that steep slopes are particularly susceptible to soil erosion due to shallow soil layers, reduced moisture, and lower nutrient availability, all of which constrain tree growth. In the northern tropical karst region, the steepest slopes are located at mid-hilltops, where prolonged solar exposure and extreme aridity result in severe soil moisture deficits, further limiting tree establishment.

Aspect primarily modulates light availability for trees, indirectly shaping heterogeneous microclimates in terms of temperature and humidity, which in turn affect tree distribution. With increasing aspect values, mingling declined, likely due to differences in sunlight exposure duration and humidity across aspects. In the northern tropical karst seasonal rainforest, hilltops receive direct solar radiation throughout the day, particularly in summer, when temperatures can exceed 60°C. Combined with high rock-bareness rate and intense evaporation, these conditions create extremely arid environments where only drought-tolerant plant species can persist. As a result, species segregation is markedly reduced on hilltops, with neighboring trees often belonging to the same species, forming simplified community structures. In contrast, enclosed depressions such as dolines buffer strong winds and accumulate rainfall, generating relatively humid microclimates. Under these conditions, species segregation is higher than on hilltops, supporting greater structural and compositional complexity.

#### 4.3.3 Rock-bareness rate

Rock-bareness generates diverse microenvironments, creating high environmental heterogeneity while exerting strong filtering effects that significantly influence plant growth and distribution. In the 15-ha forest plot, the rock-bareness rate was approximately 95% near hilltops, 85% on mid-slopes, and only 10% at the valley bottom. As rock-bareness increased, the uniform angle index exhibited an upward trend, likely because limited soil depth and poor water retention in high rock-bareness areas forced species to cluster to access nutrients and moisture. In contrast, mingling declined with increasing rock-bareness, reflecting reduced species diversity under harsher conditions.

The hilltop extreme habitat, characterized by harsh, arid conditions, filtered out less-adapted species, leaving only drought-tolerant specialists such as *Sinosideroxylon pedunculatum*, *Diospyros siderophylla*, *Pistacia weinmannifolia*, and *Boniodendron minius* which are primarily distributed on mountain tops. In contrast, species such as *Erythrina stricta*, *Vitex kwangsiensis*, *Sterculia monosperma*, and *Ficus hispida* are mainly restricted to depressions with low rock-bareness rates. Consequently, high rock-bareness at the hilltops reduced species segregation, mitigating adverse effects and enhancing survival among drought-tolerant trees. Li et al. (2022) reported that in karst regions of southwestern China, rocks altered tree size but not species diversity, suggesting a neutral role in maintaining diversity. This finding is inconsistent with our results, which may reflect the presence of rare species in high-elevation barren zones, where complex microhabitats can support higher diversity. Conversely, in highly barren areas where nutrient scarcity constrains tree establishment, diversity tends to decline. Overall, variation in rock-bareness drives differences in plant light-resource utilization and ultimately shapes tree growth and distribution patterns (Chen et al., 2014).

#### 4.3.4 Mean diameter at breast height and the number of individual plants

The increase in mingling with rising mean diameter at breast height is consistent with previous studies (Pommerening and Uria-Diez, 2017; Wang et al., 2021, 2018). The mingling-size hypothesis, a spatial extension based on the Janzen-Connell effect and the population protection effect, posits that large trees are often surrounded by smaller, heterospecific neighbors and that species mixing is positively correlated with size inequality. Our findings support this hypothesis, suggesting its applicability to karst natural forests. Generally, large trees are associated with lower diversity but higher mingling compared to small trees. However, disturbances, management practices, and other ecological mechanisms may reduce mingling as tree size increases. Density-dependent processes can further modulate neighborhood species segregation at the community level. For small trees, dispersal limitation and environmental filtering often promote conspecific clustering, whereas large trees tend to display higher mingling.

To sustain growth, large trees compete intensely for resources, leading to self-thinning and long-term reductions in the number of conspecific neighbors. The resulting mortality creates microsites favorable for heterospecific colonization, thereby promoting mingling (Li et al., 2020). However, in our study, no significant difference in mingling was observed between large- and small-diameter trees, possibly reflecting the influence of stand developmental stage or local environmental conditions (Yang et al., 2019). Contrasting evidence from Knysna forests shows a negative correlation between mingling and size inequality, indicating that high spatial mingling need not associate with large trees (Pommerening et al., 2024). The positive mingling and diameter at breast height observed here may therefore reflect context-dependent dynamics, although such trend are reported in most stands.

Mingling decreased with increasing tree abundance, consistent with observations in the Nanpanjiang Basin (Pommerening et al., 2024). Rare species are more likely to be surrounded by heterospecific neighbors, whereas abundant species exhibit lower mingling, likely due to dispersal limitation. Species with short dispersal distances tend to form clumped distributions, increasing conspecific encounters and reducing mingling, a pattern supported by previous studies on dispersal constraints (Ma et al., 2024). As a result, greater species diversity enhances tree heterogeneity and promotes more uniform distributions.

### 4.4 The response of sapling diversity to stand spatial structure

Stand spatial structure plays an important role in shaping the microenvironment and distribution of understory saplings. Our results demonstrated significant correlations between mingling and sapling diversity, identifying mingling as the primary factor driving understory diversity. Increased mingling intensity reflects greater spatial mixing among different tree species, which enhances interspecific competition. This competition leads to mutual suppression among adult trees, thereby releasing nutritional space and resources for sapling establishment. In addition, the crowns, branches, and trunks of diverse species in mixed stands form complex vertical and horizontal structures that effectively intercept and weaken wind forces while creating favorable microclimatic conditions, such as suitable light, temperature, and moisture, for sapling growth. Thus, mingling influences the species, growth, and distribution of saplings (Yang et al., 2023). Previous studies have suggested that species richness regulates the spatial distribution of trees (Bastias et al., 2020). However, this study found no significant correlation between Margalef richness index and uniform angle index. This discrepancy may reflect differences in analytical methods or site-specific environmental conditions.

## 5 Conclusions

In this study, we analyzed the dynamic characteristics of stand spatial structure and its driving factors within the framework of spatial structural units. Building on this, we further explored the effects of spatial structure on sapling diversity. Our findings highlight the ecological benefits of spatial heterogeneity in natural mixed forests at the individual level and emphasize the importance of stand spatial structure as a key driver of sapling diversity. The results suggest that the forest stand has developed into a climax community stage. Tree mortality and recruitment contributed substantially to structural changes by increasing the number of reference trees across different categories. Among the drivers examined, topographic factors exerted a stronger influence on stand spatial structure than biotic factors. Moreover, mingling was identified as the principal determinant of sapling diversity, acting through its effects on microenvironmental conditions and spatial distribution.

Despite these insights, several limitations remain. Due to the large dataset and computational memory constraints, spatial structure calculations could not be extended to individual trees with diameter at breast height < 5 cm, which may have introduced bias into the overall analysis. In addition, the evaluation of influencing factors was not fully comprehensive, as soil physicochemical properties were not incorporated. Future studies should therefore include all size classes of individuals to more accurately assess regeneration, mortality, and survival processes, while also integrating soil physicochemical attributes to provide a more complete understanding of stand dynamics. Furthermore, the interactive mechanisms linking stand spatial structure, tree growth, and their environmental drivers warrant further investigation to refine and extend the present findings.

## Data Availability

The original contributions presented in the study are included in the article/supplementary material. Further inquiries can be directed to the corresponding author/s.
